# Artificial intelligence in clinical decision‐making: Rethinking personal moral responsibility

**DOI:** 10.1111/bioe.13222

**Published:** 2023-09-19

**Authors:** Helen Smith, Giles Birchley, Jonathan Ives

**Affiliations:** ^1^ Centre for Ethics in Medicine University of Bristol Medical School Bristol UK

**Keywords:** artificial intelligence, bioethics, clinical decision‐making, clinician, ethics, responsibility

## Abstract

Artificially intelligent systems (AISs) are being created by software developing companies (SDCs) to influence clinical decision‐making. Historically, clinicians have led healthcare decision‐making, and the introduction of AISs makes SDCs novel actors in the clinical decision‐making space. Although these AISs are intended to influence a clinician's decision‐making, SDCs have been clear that clinicians are in fact the final decision‐makers in clinical care, and that AISs can only *inform* their decisions. As such, the default position is that clinicians should hold responsibility for the outcomes of the use of AISs. This is not the case when an AIS has influenced a clinician's judgement and their subsequent decision. In this paper, we argue that this is an imbalanced and unjust position, and that careful thought needs to go into how personal moral responsibility for the use of AISs in clinical decision‐making should be attributed. This paper employs and examines the difference between prospective and retrospective responsibility and considers foreseeability as key in determining how personal moral responsibility can be justly attributed. This leads us to the view that moral responsibility for the outcomes of using AISs in healthcare ought to be shared by the clinical users and SDCs.

## 1 INTRODUCTION

Healthcare is a safety critical sector, not least because there are numerous opportunities for healthcare professionals to act to influence patients′ wellbeing via clinical decision‐making and subsequent clinical intervention (or nonintervention). Patients can be inherently vulnerable ‘by virtue of their health condition and relative lack of clinical knowledge’.^1^ Even where treatment is undertaken or supervised by highly skilled clinicians, there is the risk of mistakes—either in decision‐making or in the administration of care/treatment—that may lead to patient harm. Patient care has been traditionally led by trained clinicians, who are held professionally accountable for their actions by professional codes of conduct^2^ and legally responsible by both civil and criminal law.

Clinicians assess their patient's condition and then use the outcome of that assessment to suggest treatment, from which point onward care planning is subsequently agreed with their patients. Clinical practitioners receive specialised training to perform their roles and are closely regulated to maintain standards of practice—a primary purpose of which is to protect patients from harm. Standards of practice are maintained by provisional training, followed by ongoing education and professional development. This rewards and celebrates good practice and allows sanctioning of practitioners who fall below professional standards, either by requiring remedial training and monitoring or sometimes expulsion from the profession altogether.

Before we continue, a brief note is needed to highlight our focus on the clinician user and not the patient voice. We assume in this paper that the clinician is a gatekeeper to what the patient is offered, and has freedom over how they arrive at their diagnosis and therapeutic recommendations. Patient autonomy comes in when they make an informed choice between available therapeutic options, but does not unambiguously extend to the tools the clinician uses for diagnosis and/or arriving at recommendations. Patients may, of course, wish to either embrace or avoid AI in their healthcare journey, and their ability to do this may well depend on the healthcare systems available. We take it for granted that ideally patients should be able to either accept or refuse AI use. We do not have space to consider this in depth, but note it should be the focus of other papers.

Recently, artificially intelligent systems (AISs) have been created by software developing companies (SDCs) to aid clinical decision‐making. Currently, these systems need not go near a patient; they merely process data input about the patient in question and generate outputs that advise the attending clinician. One well‐known example of this is IBM's ‘Watson for Oncology’. This system examines a patient's medical record, processes the information therein, calculates the patient's options and then presents the requesting clinician a rank‐order list of treatments.^3^ The putative advantages of this kind of system are that:

a) it is able to access and process complex information at a much faster rate than any human clinician whilst
b) staying entirely up to date with the most recent evidence (which is released at a volume and rate that is challenging for clinicians to comprehensively keep up with)^4^ and
c) draws down on all of that data to generate bespoke plans for a patient.

Such a system creates the potential for clinicians to make efficiencies in information management, enabling access to up‐to‐date knowledge (e.g., clinical studies), freeing up time which leads to more capacity for patient care and maybe even the opportunity to reduce clinician burnout. Any rejection of AI is therefore subject to consideration of opportunity cost (although this will not be the focus of our discussions).

Watson is clearly designed to directly influence clinical decision‐making as its usefulness resides only in it being able to operate beyond human cognitive abilities and draw conclusions that human clinicians cannot. However, an unnamed IBM Executive Consultant has been noted saying that ‘Watson does not make decisions on what a doctor should do. It makes recommendations based on hypothesis and evidence based [sic]’.^5^ This clearly frames the clinician as the decision‐maker and the person who has ultimate responsibility for the decision. This is generally not a problem when things go right, but if something goes wrong and a patient is harmed as a result of following (or not following) the AIS advice, the clinician is framed to be wholly and solely ethically responsible.

This is clearly advantageous for SDCs. Their system delivers a recommendation, and the clinical user carries the professional burden of the consequences of using that recommendation or not. The clinician is thus positioned to protect the SDC, acting as a ‘moral crumple zone’^6^—a term that describes how the balance of responsibility for actions is misattributed to particular actors when others are also involved.^7^ The clinician in this role insulates the SDC from the consequences of any of their system's errors. In this paper, we try to show that this arrangement is fundamentally wrong and that responsibility ought to be shared between clinicians and SDCs. Before we move on to the argument proper, we need to briefly respond to two brief arguments that might suggest this issue is not important.

First, it might be argued that it does not matter who is liable so long as any harm to the patient is remedied or appropriately compensated. Individual clinicians will usually be shielded from a negligence claim by their relationship with an institutional entity through vicarious liability and so will not have to personally pay. This fails to work on a few fronts, however. First, the fact that a clinician is protected financially by vicarious liability does not mean that the individual is not harmed in many other ways—both professionally and personally—by a lawsuit. Second, notwithstanding who actually pays, the status quo is imbalanced in a way that favours SDCs and presents no incentive to ensure that their systems work beyond what is required for profitability. We know that corporations may, in principle, be willing to market systems with known problems and accept the cost of legal action because that is more profitable (see Lee & Ermann's article^8^ for an example of this in the automotive industry), and it would certainly be more profitable to market an imperfect system when there is no possibility of being liable for erroneous outputs. Professional regulation of clinicians provides a mechanism to weed out poor clinical practice,^9^ but there is no comparable mechanism for SDCs.

Second, it could be argued that the clinician is rightly positioned as the final decision‐maker and bears ultimate moral responsibility and legal liability. Although many factors (e.g., colleagues or computers) may provide input and insight into a clinical decision, the clinician in charge is responsible for collating that information, sorting it, processing it, and making the final care management decision. The clinician in charge is ‘in charge’ for just that reason. Their role is to make a judgement and to identify error in any advice they are given. For that reason, if they do not spot the error, or decide to act on bad advice, they are responsible.

However, this rests on the premise that the clinician in charge is able to spot that an AIS has erred and identify an inappropriate recommendation. When advice comes from human colleagues, it is possible to understand and interrogate their reasoning: all have access to the same information and are on a level cognitive playing field. When, however, the AIS in question is not explicitly designed with the capability to meaningfully explain its reasoning, its processes and outputs can be opaque to users and not open to scrutiny. AISs employing machine learning could be an example of this: their outputs would be considerably more challenging for clinicians to evaluate, as the AIS's functionality—and therefore its outputs—would evolve over time as it learns. This means that the recommendation offered by an AIS today could very well be different tomorrow, even if the patient's circumstances were unchanged. Yet, an AIS would be only employed because it can process information in a way that the clinician cannot and draw conclusions at speed from masses of inputs that a clinician could not hope to replicate.

It is unclear how a clinician could confidently spot an error in a system that is only being used because it supposedly has superior data processing and decision‐making capacity and therefore would have the ability to arrive at conclusions that the human mind would or could not. It is not, therefore, a straightforward matter to second guess the system. A clinician would know that in not following a recommendation they did not feel was correct, they may just as easily be making a mistake because they had not spotted something that the AIS has. The same point holds true, though perhaps to a lesser extent, with systems that merely present probabilities for consideration rather than specific recommendations. If those probabilities are calculated in an opaque way and are supposed to be accurate and more reliable than could be calculated by a human clinician, then they will be informing decision‐making in exactly the same way as a system that makes recommendations.

Making this decision, when we know the AIS ‘advisor’ should be superior, but might be fallible, is no easy task. This does raise the question of whether clinicians should be routinely trained to understand and use AISs, which would serve to demystify it, aid understanding and therefore potentially reduce the potential for harm arising from its use. This would certainly be beneficial and essential as AI use becomes routine. This is no different from clinicians needing to undertake training and achieving competence prior to using any new tool or technique.^10^ Indeed, Health Education England has developed a basic framework regarding AI knowledge to that effect,^11^ although we would draw attention to the challenges of adding yet more to an already very crowded curriculum. We also note that even with this acquired knowledge, the unpredictability and accompanying uncertainty about the accuracy of AIS outputs will ever remain. This uncertainty means the clinician will face making the wrong decision by either following the system (if it turns out to be wrong) or not following it (if it turns out to be right), and they have no way to understand the reasoning behind a recommendation because it will be inaccessible.

Of course, training will only make a difference if the systems are in principle understandable, and it might be argued that our argument sets up a straw man in assuming they are not or cannot be. Rudin,^12^ for example, calls for any AI used in high‐stakes decisions to either be interpretable or be accompanied by post hoc models that explain the AIS's output. Indeed, AISs that can give ‘clear and adequate information’^13^ to users will be a requirement of the anticipated European Union's AI Act,^14^ and if it is possible for system to do this—to be both adequately transparent and understandable to the clinician—then our argument will have less bite.

However, Rudin also notes that ‘[b]lack box machine learning models are currently being used for high‐stakes decision making throughout society, causing problems in healthcare, criminal justice and other domains’,^15^ suggesting that this interpretable ideal is not yet attainable. We are sceptical that it will ever be. Given that AIS development is ongoing and the resulting systems increasingly complex, there will always be a point where an output's intricacy is beyond a reasonable level of understanding that can be expected of a human user. Whilst a user can be given clear information, the notion of ‘adequacy’ will be a constantly moving target and a challenge to realise. Too little information fails to educate the AIS user, so more information is desired. But as systems become more complex, the volume of information that users must be presented with in order to understand AIS outputs will increase. Vast amounts of information will become effectively opaque to users as it is unreasonable to expect them to internalise, process and understand all of the information that has been put in front of them.

Given this, we argue that using the clinician as a moral crumple zone is unjust. The SDC might not be at the bedside when their AIS is used, but the SDC should not be insulated from responsibility for an error arising directly from an AIS they have created, situated in a clinical space and marketed on the basis that it can do things clinicians cannot.

SDCs are new actors in clinical decision‐making, and they arguably do have a role to play. If AISs are to be encouraged, deployed and adopted in the clinical decision‐making space, consideration needs to be given to how responsibility for harms arising from their use might be allocated to the various actors when these systems are used at the point of care. Failing to appropriately allocate personal moral responsibility to the correct actors is itself a moral wrong.

Furthermore, taking away the moral crumple zone that SDCs currently enjoy would encourage them to think more about the safety of their product, as well as address the morally problematic imbalance of clinicians carrying all the burden of responsibility—which is also a significant disincentive to their adoption.

In what follows, we will explore factors that must be considered when thinking about the allocation of personal moral responsibility for the outcomes of AIS use between the SDC and the clinician.

## 2 HOW CAN WE ASSIGN PERSONAL MORAL RESPONSIBILITY?

The full debate surrounding personal moral responsibility is complex;^16^ thus, discussion shall be limited only to this paper's concerns. Let us start by defining personal moral responsibility, and then we can identify two distinctions that allow moral responsibility to be assigned to SDCs.

Personal moral responsibility is the individual's obligation or duty to ensure that something is done or obtained, and an individual's moral burden is attached to them due to the role that they perform within the context being discussed.^17^ Personal moral responsibility is envisaged in this definition as being assigned to individuals, but groups may also have personality, and those sharing the same characteristics, aims, values and goals (which are ‘personal’ to that organisation) might be awarded ‘personal’ moral responsibility similar or identical to that of individuals. Employing Beauchamp and Childress′^18^ principle of nonmaleficence, it is reasonable to assume that the obligation of one person to another would, at the very least, ensure that the first did not harm the second. This could be extended to beneficence, where one ought to prevent/remove evil or harm and promote or do good^19^; ergo, obliging the first person to contribute to the second's welfare.

Moral responsibility might be considered as ‘professional’ rather than ‘personal’ when considering the actions of clinicians; yet, if the clinician is forced to act without professional guidance—which they will be until professional guidance around AI use is issued for clinicians—they are acting at an individual level using their personal judgement, rather than in unison with their colleagues as per a proscribed professional standard. Furthermore, the distinction between professional and personal responsibility may not be as stark as might first appear. Whilst professional responsibility holds for members of specific professions, individual professionals can still be considered to have personal moral responsibility to ensure that they fulfil professional moral responsibilities. As such, there is an entanglement here that we feel permits us to speak of clinicians having personal moral responsibility in a professional setting.

Personal moral responsibility can be broken down into two types^20^:

1) Prospective personal moral responsibility can be characterised as a forward‐looking duty. A clinical duty of care is an example of prospective responsibility, wherein a clinician is considered responsible for looking after the health interests of another person.
2) Retrospective personal moral responsibility can be characterised as backward‐looking accountability. Here, past actions/omissions are judged against some standard, and if that standard is not met, one is held responsible for the harm eventuating from the action/omission.

Regulation of clinical professions exemplifies the adoption of both prospective and retrospective responsibility. Prospective responsibility is evidenced by the development of each of the professional bodies’ codes of conduct, and retrospective responsibility is demonstrated by the adoption of ‘fitness to practice’ hearings to enforce those codes and hold clinicians accountable for breaches. A negative outcome for the professional could include removal from their profession's register, which prevents the clinician from taking up further clinical work,^21^ thus providing a mechanism for the regulator to prospectively prevent patient harm.

Whilst personal moral responsibility is embraced by the clinical professions, SDCs do not have a comparable established professional duty of care and are not professionally regulated. Accordingly, SDCs are not held to account for their actions by their profession nor penalised by a regulator should they fail to take care, but they could, in theory, be penalised through private legal action brought by affected patients. It is important, therefore, to consider whether there are any good grounds to hold SDC accountable for harms arising from the presence of the technology in a clinical space. To do this, we will further unpack prospective and retrospective personal moral responsibility.

### 2.1 Prospective personal moral responsibility

SDCs are attempting to influence patient care by creating and deploying systems that they claim can aid the clinician in their decision‐making. If the SDC is situating themselves in a position of clinical influence and aiming to affect the clinical decision‐making space, there is a clear intention for the AIS to directly affect the patient to whom the decision‐making relates. Consequently, we argue, SDCs should be similarly prepared to adopt the clinician's position of prospective personal moral responsibility towards patients—a duty of care.

### 2.2 Retrospective personal moral responsibility

If we accept that SDCs have duty of care similar to clinicians, owing to their intention to affect clinical decision‐making, then if that duty of care is breached, then SDCs should also bear moral responsibility for any adverse events that follow from that breach. Responsibility to deal with the outcomes of adverse events is encompassed in retrospective responsibility. In fulfilling prospective personal moral responsibility, the conditions are created for an SDC to be legally liable. Liability and duty of care are terms that are commonly used in legal mechanisms of liability; yet, to be clear, this paper focusses on ethics and governance. If an SDC's duty of care is proven, their prospective responsibility means that they need to think carefully about what the foreseeable consequences of the deployment of their AIS would be.

Retrospective personal moral responsibility is not concerned with a duty that is yet unfulfilled (i.e., a duty of care) but with harm arising from failing to fulfil a duty owed to another.^22^

Retrospective responsibility is established when two conditions are fulfilled. As per Fuscaldo,^23^ a person is responsible to the consequence of an action if (and only if):

1. the agent was free to act otherwise/acted voluntarily, and;
2. the consequences of the action were reasonably foreseeable.

Fuscaldo holds that if a reasonable person could foresee the consequences that follow an action, then the ‘reasonably foreseeable’ criterion is met.^24^

Regarding the first condition, to be morally responsible, the actor must be acting voluntarily, and they must be freely able to act otherwise. We acknowledge the vast literature around the concept of freedom of action, but for the purposes of this paper, let us assume that SDCs are not being forced to make systems to be deployed for use in clinical settings, and they could choose not to. If this much can be accepted, they meet the voluntariness condition for moral responsibility. Similarly, the clinician appears free to not use the system that the SDC is offering and so also appears to meet this criterion. However, there could be factors that may restrict this freedom, particularly if there are professional or patient demands on clinicians to make use of all the tools that are at their disposal, including AISs.

One such example could be if an authoritative body such as the U.K.'s National Institute for Clinical Excellence (NICE) advises that an AIS may be used. Due to this, and on the understanding that it is both beneficial and cost‐effective, a hospital buys access to the AIS with the expectation that it will be used. The clinician's medical practice insurance expects the AIS to be used if the hospital has made it available, and the patient has demonstrated a wish for the AIS to advise their care. In this scenario, the clinician experiences multifaceted pressures that may reduce their ability to resist using the AIS. Whilst they may be professionally expected to provide a good standard of practice and care,^25^ which may involve rejecting AIS use if they judge necessary, arguably, the clinician has more voluntariness restricting factors acting on them than the SDC.

Regarding the second condition, it is certainly foreseeable that an AIS could make an error, or that a clinician could make an error in using it, that could lead to patient harm, and so that criteria also appears to be met.

Here is a hypothetical example to illustrate. Caruana et al.^26^ described the design of a system (which was never deployed to healthcare) that could predict the probability of death for patients with pneumonia; high‐risk patients could be admitted to the hospital whilst low‐risk patients were treated as outpatients. This kind of system would have the potential to be useful for bed management and helping to predict and direct the most appropriate resources to each patient, which could be very useful in a resource‐challenged environment such as healthcare.

The research team had noted that an asthmatic with pneumonia is normally aggressively treated and sent to the intensive care unit, and because critical care treatments are so effective, an asthmatic's risk of dying from pneumonia is lower than the rest of the population. But the system trained on this data interpreted it differently and determined that if a patient with pneumonia had asthma, then the presence of asthma lowered the risk of death.

This system was not able to take into account the contextual information that comes with the care of complex asthmatic patients—that high support interventions are needed for an asthmatic with pneumonia to have a good outcome. If this AIS were deployed and a clinician uncritically accepted the AIS's risk analysis, then there is the potential that an asthmatic patient with pneumonia could be sent home when a critical care intervention was needed to avoid foreseeable catastrophic consequences. It would be hard to view this as an unforeseeable event, especially in the presence of a clinical professional who should have known to escalate care; however, if this system were deployed, it would not be entirely implausible for this kind of mistake to be made during a period of busy winter‐pressures upon an already resource‐restrained clinical environment. Were there an absence of foreseeability, it might be argued that just because an actor has causally contributed to a consequence does not necessarily mean that they are morally accountable for it. But, in this scenario, personal moral responsibility could plausibly be jointly allocated to both the clinician and the SDC, as neither would have ensured that that patient had been correctly identified as at high risk of death.

Given we have established that it is plausible for an SDC to have both prospective and retrospective responsibility, we can consider how those responsibilities might be fulfilled.

To take prospective responsibility first, an SDC is obliged to think about the negative and/or harmful consequences that could result from the operation of their system in a clinical environment and take steps to mitigate against them. The SDC would need to rationally consider what is known about the likely consequences of their system being used in the clinical environment and articulate how their decision to deploy their system was reached. They would then need to demonstrate that they had acted to mitigate the risks of harm that were identified or explain why some risks are so small they require no action.

The overarching duty of care (prospective responsibility) requires the possible consequences of AIS use to be carefully considered by the SDC. In doing this, the SDC can show that they acted rationally and reasonably, but in doing so, they both set up and meet the conditions of the foreseeability criterion. This then makes them prima facie liable for retrospective moral responsibility.

Of course, in taking their prospective moral responsibility seriously and mitigating against foreseeable risk of harm, they may well also protect themselves against liability from any foreseen harms that do eventuate.

If an unforeseen harm eventuates, and because it was unforeseen, no steps were taken to mitigate against it, the question then becomes whether the harm *should* have been foreseen. This is where the test of reasonable foreseeability kicks in. If, after the fact, the harm is considered to be something that a reasonable, rational and diligent actor could and should have foreseen, then the fact that the SDC failed to foresee it would not matter—they can still be held morally responsible for harm.

The existence of a prospective moral responsibility means that the SDC has a duty of care to mitigate against the eventuation of foreseeable risks of harm. One way an SDC may choose to do this is by warning the user of the risk of an erroneous output that could be harmful to a patient. This is essentially a ‘user beware’ warning.

In this way, the SDC shifts the burden of personal moral responsibility onto the clinical user. This does not seem ethically justifiable given that it is reasonably foreseeable that users of the system are, and will be, imperfect. It is, of course, fair to hold a clinician personally morally responsible for the harmful consequences of a reckless decision or when a glaringly dangerous AIS recommendation is followed. However, when the AIS is incorrect, and the mistake cannot reasonably be detected or understood—especially in the context of a clinician using a system that is putatively superior in its ability to process information—then it is wholly unfair to allocate *all* responsibility to the clinical user. In simply stating ‘user beware’, the SDCs may discharge some of their personal moral responsibility, but not all; some must remain due to their AIS's ongoing presence in the clinical decision‐making space and the fact that it is clearly foreseeable that clinicians are not perfect gatekeepers.

If (i) a system is designed to influence clinical decision‐making, (ii) it is foreseeable that an AIS may dispense a harmful output, and (iii) it is also foreseeable that a clinician might unwittingly accept and use that output, then the ‘reasonably foreseeable’ criterion is met for the SDC. Thus, a freely acting SDC will be morally responsible for many of the foreseeable consequences of those faulty outputs that their voluntarily deployed AIS dispenses. This does not relieve the clinician of their moral responsibility for harm that occurs from their using an AIS, but it does create space for the SDC to join the clinician and share in that moral responsibility.

The primary reason for the SDC not being able to pass on all the moral responsibility to the clinician in charge is the opacity of their system. As noted above, an AIS that is sophisticated enough to be worth having in a clinical space may be so complex that it operates as a black box and not be understandable by, or explainable to, the clinician. In some applications of machine learning, it will be utterly impossible to understand how the AIS reaches its conclusions. As described earlier in this paper, the issue with opaque AISs deployed for clinical decision‐making is that the outputs are not always predictable, as they will change as the system learns with each new experience and then applies that learning to each subsequent use. This means that the AIS's specific outputs may not always be foreseeable. And that is, of course, the whole point, as if its outputs could be foreseen it would not be needed. They are, however, *foreseeably unforeseeable*, and this uncertainty presents a foreseeable (if probably relatively small) risk of patient harm.

The SDC could still, however, argue that they have designed the AIS to be implemented in conjunction with a clinical decision‐maker, who is there precisely in order to spot outputs that could be harmful. In doing so, they have devolved the personal moral responsibility for the consequences of using the AIS to the clinician who is using them for clinical decision‐making, and again the SDC uses the clinician as a moral crumple zone.^27^ But the fact that the AIS is opaque, and is provided for use by the clinician on the grounds that is able to process information, and reach conclusions, that the clinician cannot, means that the clinician may not reasonably be expected to take on that role of safety gatekeeper, because they are placed in a no‐win dilemma.

They are warned that the system is not perfect, and so its outputs cannot be relied upon and should be double‐checked and confirmed, but they are simultaneously told that the system can do things, and spot things, that the clinician cannot. The clinical gatekeeper is essentially told that the AIS is a superior clinical decision‐maker, until it is not, but because the system is opaque, its reasoning cannot be scrutinised and so it is impossible to know for sure when an unforeseen output is correct or incorrect. In this case, if the clinician follows the erroneous output and the patient is harmed, then they are at fault, but if they do not follow a correct output and the patient is harmed, then they are also at fault—and they have no way of discerning whether the system output is erroneous or not.

If a correct system output is not guaranteed, and if the clinician is not able to scrutinise the output to determine if is it safe or correct, then patient safety is at risk. If the clinician is to be held entirely responsible for any harms that follow, then the clinician would then be justified in not using the AIS at all, as that would be the only way to (a) fulfil their duty of care to maintain patient safety and (b) protect themselves professionally. This would, in turn, mean that the patient community can never benefit from AIS in a clinical setting.

Given this, one solution would be for an SDC to simply withhold AIS systems until they are safe enough to be deployed without the need for clinical oversight. This, however, is unlikely to be feasible, not least because these systems need to be deployed in order to learn and become more accurate. It is also unlikely to be considered cost‐effective to wait until a system can be error‐free before deployment, and, further, error‐free perfection seems like a very high bar to have to meet—and would certainly hold the system to a higher standard than we hold clinicians.

Clinicians are likely to welcome any technological innovation that helps patients, and it would not be unreasonable for clinicians to welcome beneficial AISs even if they are not perfect. If, however, the price of AIS use is unequal, burdensome, and unjust allocation of personal moral responsibility for errors that are beyond their control or understanding, that might be enough reason for the clinical professions to reject AIS use entirely.

It is reasonable to suggest, then, that addressing this injustice will be an essential component of ensuring that the putative benefits of AISs in clinical spaces can be reaped. The way to do this is likely to be to ensure that moral responsibility—both prospective and retrospective—is shared by all relevant actors.

We have already argued that prospective and retrospective responsibility can be allocated to SDCs, and we have further provided an argument to show that it is in the SDCs' own interest to accept this responsibility. Finally, we will consider how this responsibility might be formalised.

A simple answer could lie in developing sector‐specific regulation for SDCs and professional regulation for SDC employees who contribute to the development of clinical AISs. This would put them on a par with the professional regulation of clinicians and would formalise the prospective duty of care and create space for professional disciplinary proceedings if professional standards are not met. However, it would be near impossible to answer questions around determining which technologist had contributed to which part of the AIS that had resulted in it offering a harmful recommendation.

As Taddeo and Floridi^28^ note, existing responsibility frameworks tend to focus on the actions of individuals and are thus unsuited to situations where many actors are involved in a complex design and production process. This is problematic not only because it is likely impossible to isolate single responsible agents but also because it is unlikely to be useful or helpful to isolate a specific individual as culpable, because that individual is unlikely to be able to fully compensate for harm experienced by a patient. A group model of responsibility could be practicable here, where (as described earlier) the SDC is considered to have a group‐level personality comparable to that held by individuals.

## 3 A SHARED MODEL OF RESPONSIBILITY

We have so far described a fragmented approach to personal moral responsibility where each party acts as an individual trustee representing their own interests. Given that the allocation of individual personal moral responsibility arguably results in injustice for clinicians which, as well as being a wrong in itself, does not seem to be in the interests of SDCs, we follow Whitby in calling for the development of a *shared* model of responsibility in this sector.

Whitby^29^ argues that ‘[i]f an AI system gives inappropriate advice then its designers and builders must share responsibility for any unfortunate medical consequences’. To further specify this idea, we suggest that it is incumbent on the SDC to accept their share of moral responsibility, alongside clinicians and clinical institutions, and acknowledge their liability to share the costs if something goes wrong. Rather than searching for a specific actor to censure, a shared model would focus on both ensuring appropriate recompense to the affected patient, and preventing harms and enhancing safety,^30^ which would reflect the contemporary healthcare movement of ‘learn not blame’.^31^

Sharing responsibility would need novel collaborative working practices from the outset of AIS design. At a minimum, a common working platform for involved actors would be needed to tackle the causes of foreseeable harms that could eventuate from AIS use. Such a shared platform may afford the opportunity for meaningful discussion between actors in the clinical decision‐making space, resulting in clarity on practical issues such as AIS accuracy or the potential for atrophy of vigilance^32^ (i.e., clinicians coming to trusting AISs uncritically when they know that they should not).

This is not to say that AISs are being created in a vacuum with no clinical input. For example, Watson was developed with the clinical teams of Memorial Sloan Kettering,^33^ and SDCs are certainly developing their technologies with clinicians on their teams. Rather, our point is that there is a need for clinical users, external to the SDC and not directly involved in development, to have the opportunity to examine the AISs. This would include subjecting them to third‐party critical review and clinical trials to assess effectiveness prior to deployment,^34^ and for that external review to be reported transparently for wider clinical appraisal.

Indeed, ensuring that independent clinicians can communicate with SDCs, and vice versa, at the points of development, deployment and beyond, has the potential for many benefits. On an ethical level, it may allow clinicians to articulate the injustice of carrying the burden of sole responsibility when using AISs, and it will give the SDC an opportunity to acknowledge the injustice and understand the impact it could have on their technology being implemented. It is in the interest of SDCs for clinicians to have confidence in, and make use of, their technologies—and thus SDCs might recognise voluntarily a shared interest in demonstrating that they themselves are confident in, and committed to the safety of, their technologies and this can be shown via actions such as encouraging external scrutiny of their AISs.

SDCs additionally can demonstrate that they are a trustworthy actor in this space by recognising, accepting and embracing the idea they are in this together with clinicians and patients. The siloed thinking of ‘a clinician makes the final decision when using an AIS and therefore takes all responsibility for the consequences of that decision’ must be abandoned. Instead of a finger‐pointing exercise, shared responsibility for the use of AISs from the outset of AIS development would invoke principles of problem‐solving through collectively building assurances of safe AIS development, deployment and use, as well as developing robust plans for restitution if/when harms occur (e.g., developing and employing insurance schemes such as risk pooling).^35^

Importantly, this approach does not advocate a flipped scenario whereby clinicians are able to misattribute blame to AISs every time something goes wrong. Rather, shared responsibility enables each actor to carry the weight of their own actions. In the case of SDCs, their actions would be to have contributed to the knowledge that informed the harm and the clinicians would have contributed by making a decision that enacts harm. Subsequently, the proportion of responsibility attributed to each actor will vary as per the retrospective examination of each incident after the fact, ideally informed by jointly developed and agreed criteria before deployment.

Yet, shared responsibility is one potential answer to what is an immensely complex issue. The question of how to distribute responsibility will admit more than one correct solution and the aim of this paper is to outline one kind of solution that ought to be considered. Our arguments pertain to an as yet speculative future, as AISs are not as yet commonplace in healthcare, and as we learn more about how they can be, and are used, we will understand better how to regulate them. This of course should not stop us from considering plausible futures and outcomes now.

## 4 CONCLUSION

We have discussed the application of prospective and retrospective personal moral responsibility to both clinicians and SDCs when considering the use of AIS in the clinical decision‐making environment. Our discussion has provided grounds for both the clinician and the SDC to shoulder a prospective duty of care to the patient for whom the AIS is to be used. If there is a failure in that duty of care, there might be grounds for retrospective personal moral responsibility to be justifiably allocated to either or both the clinician and the SDC, dependent upon the circumstances.

Responsibility can be allocated fairly to both clinicians and SDCs; we have suggested that a shared model of responsibility is appropriate and should be agreed before any deployment or use of AISs in clinical decision‐making. Without this, an unjust burden of personal moral responsibility will likely be borne by clinical users.

## CONFLICT OF INTEREST STATEMENT

The authors declare no conflicts of interest.

## Footnotes

^1^
Smith, H., Fotheringham, K. (2020). Artificial intelligence in clinical decision‐making: Rethinking liability. *Medical Law International*, *2*0(2), 131–154, 133.

^2^
Health & Care Professions Council. (2016). *Standards of conduct, performance and ethics*. https://www.hcpc-uk.org/publications/standards/index.asp?id=38; Nursing and Midwifery Council. (2018). *The code for nurses and midwives*. https://www.nmc.org.uk/standards/code/read-the-code-online/; General Medical Council. (2019). *Good medical practice*. https://www.gmc-uk.org/ethical-guidance/ethical-guidance-for-doctors/good-medical-practice

^3^
Luxton, D. D. (2019). Should Watson be consulted for a second opinion? *AMA Journal of Ethics*, *21*(2), E131–E137. https://doi.org/10.1001/amajethics.2019.131

^4^
Alper, B. S., Hand, J. A., Susan E. G., Kinkade, S., Hauan, M. J., Onion, D. K., & Sklar, B. M. (2004). How much effort is needed to keep up with the literature relevant for primary care? *Journal of the Medical Library Association*, 92(4), 429–437.

^5^
Hengstler, M., Enkel, E., & Duelli, S. (2016). Applied artificial intelligence and trust—The case of autonomous vehicles and medical assistance devices. *Technological Forecasting & Social Change*, *105*, 105–120, 115. https://doi.org/10.1016/j.techfore.2015.12.014

^6^
Elish, M. C. (2019). Moral crumple zones: Cautionary tales in human‐robot interaction. *Engaging Science, Technology, and Society*, *5*, 40–60, 2. https://doi.org/10.17351/ests2019.260

^7^
Ibid.

^8^
Lee, M. T., & Erman, M. D. (1999). Pinto “madness” as a flawed landmark narrative: An organizational and network analysis. *Social Problems*, *46*, 30–47. https://doi.org.10.2307/3097160

^9^
Health & Care Professions Council. (2018). *Fitness to practise*—*Raising concerns*. https://www.hcpc-uk.org/concerns/raising-concerns/; Nursing and Midwifery Council. (2018) *Our investigations*. https://www.nmc.org.uk/ftp-library/understanding-fitness-to-practise/investigations/; General Medical Council. (2022) *Investigating and acting on concerns about doctors*. https://www.gmc-uk.org/about/what-we-do-and-why/investigating-and-acting-on-concerns-about-doctors

^10^
NMC. (2015, January 29). *NMC code of conduct*. https://www.nmc.org.uk/standards/code/read-the-code-online/; GMC. (2019, April 29). *Good medical practice*. https://www.gmc-uk.org/ethical-guidance/ethical-guidance-for-doctors/good-medical-practice/domain-1---knowledge-skills-and-performance#develop-and-maintain-your-professional-performance; HCPC. (2016, January 26). *Standards of conduct, performance and ethics*. https://www.hcpc-uk.org/standards/standards-of-conduct-performance-and-ethics/

^11^
Health Education England. (2023). *Artificial Intelligence (AI) and Digital Healthcare Technologies Capability framework*. https://digital-transformation.hee.nhs.uk/building-a-digital-workforce/dart-ed/horizon-scanning/ai-and-digital-healthcare-technologies

^12^
Rudin, C. (2019). Stop explaining black box machine learning models for high stakes decisions and use interpretable models instead. *Nature Machine Intelligence*, *1*, 206–215. https://doi.org.10.1038/s42256-019-0048-x

^13^
European Commission. (2022). *Regulatory framework proposal on artificial intelligence*. https://digital-strategy.ec.europa.eu/en/policies/regulatory-framework-ai

^14^
Ibid.

^15^
Rudin, op cit. note 12, p. 206.

^16^
Fuscaldo, G. (2006). Genetic ties: Are they morally binding? *Bioethics*, *20*, 64–76. https://doi.org/10.1111/j.1467-8519.2006.00478.x

^17^
Zimmerman, M. J. (1992). Responsibility. In L. Becker & C. Becker (Eds.), *Encyclopaedia of ethics. Volume II:L‐Index* (pp. 1089–1095). Garland Publishing Inc.

^18^
Beauchamp, T., & Childress, J. (2019) *Principles of biomedical ethics* (8th ed.). Oxford University Press

^19^
Ibid.

^20^
Zimmerman, op. cit. note 17.

^21^
Health & Care Professions Council, Nursing and Midwifery Council, General Medical Council, op. cit. note 9.

^22^
Zimmerman, op. cit. note 17.

^23^
Fuscaldo, op. cit. note 16.

^24^
Ibid.

^25^
GMC. (2019). *Good medical practice*. https://www.gmc-uk.org/ethical-guidance/ethical-guidance-for-doctors/good-medical-practice/domain-1---knowledge-skills-and-performance#apply-knowledge-and-experience-to-practice

^26^
Caruana, R., Yin, L., Gehrke, J., Koch, P., Sturm, M., & Elhadad, N. (2015). *Intelligible models for healthcare: Predicting pneumonia risk and hospital 30‐day readmission*. KDD '15: Proceedings of the 21th ACM SIGKDD International Conference on Knowledge Discovery and Data Mining. https://doi.org/10.1145/2783258.2788613

^27^
Elish, op. cit. note 6.

^28^
Taddeo, M., & Floridi, L. (2018). How AI can be a force for good. *Science*, *361*(6404), 751–752.

^29^
Whitby, B. (2015). Automating medicine the ethical way. In S. P. van Rysewyk & M. Pontier (Eds.), *Machine medical ethics (intelligent systems, control and automation: Science and engineering)* (pp. 223–232, 229). Springer.

^30^
Ibid.

^31^
Robinson, F. (2019). Learn not blame: How a grassroots campaign struck a chord. *BMJ*, *365*, l4232.

^32^
Life Healthcare Communications. (2019). *What are you doing, Dave?* http://www.life-healthcare.com/life-blog/what_are_you_doing_dave/

^33^
Ross, C., & Swetlitz, I. (2017). *IBM pitched its Watson supercomputer as a revolution in cancer care. It's nowhere close*. STAT. https://www.statnews.com/2017/09/05/watson-ibm-cancer/

^34^
Ibid.

^35^
Smith & Fotheringham, op. cit. note 1.

